# Racial and Ethnic Digital Divides in Posting COVID-19 Content on Social Media Among US Adults: Secondary Survey Analysis

**DOI:** 10.2196/20472

**Published:** 2020-07-03

**Authors:** Celeste Campos-Castillo, Linnea I Laestadius

**Affiliations:** 1 Department of Sociology University of Wisconsin-Milwaukee Milwaukee, WI United States; 2 Zilber School of Public Health University of Wisconsin-Milwaukee Milwaukee, WI United States

**Keywords:** COVID-19, digital divides, user characteristics, race, ethnicity, algorithm bias, social media, bias, surveillance, public health

## Abstract

**Background:**

Public health surveillance experts are leveraging user-generated content on social media to track the spread and effects of COVID-19. However, racial and ethnic digital divides, which are disparities among people who have internet access and post on social media, can bias inferences. This bias is particularly problematic in the context of the COVID-19 pandemic because due to structural inequalities, members of racial and ethnic minority groups are disproportionately vulnerable to contracting the virus and to the deleterious economic and social effects from mitigation efforts. Further, important demographic intersections with race and ethnicity, such as gender and age, are rarely investigated in work characterizing social media users; however, they reflect additional axes of inequality shaping differential exposure to COVID-19 and its effects.

**Objective:**

The aim of this study was to characterize how the race and ethnicity of US adults are associated with their odds of posting COVID-19 content on social media and how gender and age modify these odds.

**Methods:**

We performed a secondary analysis of a survey conducted by the Pew Research Center from March 19 to 24, 2020, using a national probability sample (N=10,510). Respondents were recruited from an online panel, where panelists without an internet-enabled device were given one to keep at no cost. The binary dependent variable was responses to an item asking whether respondents “used social media to share or post information about the coronavirus.” We used survey-weighted logistic regressions to estimate the odds of responding in the affirmative based on the race and ethnicity of respondents (white, black, Latino, other race/ethnicity), adjusted for covariates measuring sociodemographic background and COVID-19 experiences. We examined how gender (female, male) and age (18 to 30 years, 31 to 50 years, 51 to 64 years, and 65 years and older) intersected with race and ethnicity by estimating interactions.

**Results:**

Respondents who identified as black (odds ratio [OR] 1.29, 95% CI 1.02-1.64; *P*=.03), Latino (OR 1.66, 95% CI 1.36-2.04; *P*<.001), or other races/ethnicities (OR 1.33, 95% CI 1.02-1.72; *P*=.03) had higher odds than respondents who identified as white of reporting that they posted COVID-19 content on social media. Women had higher odds of posting than men regardless of race and ethnicity (OR 1.58, 95% CI 1.39-1.80; *P*<.001). Among men, respondents who identified as black, Latino, or members of other races/ethnicities were significantly more likely to post than respondents who identified as white. Older adults (65 years or older) had significantly lower odds (OR 0.73, 95% CI 0.57-0.94; *P*=.01) of posting compared to younger adults (18-29 years), particularly among those identifying as other races/ethnicities. Latino respondents were the most likely to report posting across all age groups.

**Conclusions:**

In the United States, members of racial and ethnic minority groups are most likely to contribute to COVID-19 content on social media, particularly among groups traditionally less likely to use social media (older adults and men). The next step is to ensure that data collection procedures capture this diversity by encompassing a breadth of search criteria and social media platforms.

## Introduction

### Background

Amid the novel coronavirus disease (COVID-19) pandemic of 2020, US adults are turning to social media to consume and share information [1]. Public health surveillance experts are already leveraging the data created by social media users to track the spread and effects of COVID-19 across populations [2-6]. Sets of social media data are also appearing in open access repositories to facilitate this work [7]. In light of COVID-19, several scientific journals are fast-tracking the peer-review process, creating an unprecedented pace of publication for new social media surveillance studies [8,9]. This in turn is accelerating the availability of analyses that can be used to inform interventions and policies aimed at combating the direct and indirect effects of COVID-19 in society.

Despite the potential benefits of social media data in providing real-time insight into COVID-19, important limitations remain. As noted in prior studies, representativeness is an ongoing challenge for social media surveillance systems [10-12]. Among the factors shaping the representativeness of social media data are racial and ethnic digital divides [13-15], in which members of racial and ethnic minority groups are less likely to access the internet and use social media [16]. In turn, the absence of social media data from racial and ethnic minority groups may bias findings from social media surveillance systems. In the context of the COVID-19 pandemic, biases due to the underrepresentation of social media data from members of racial and ethnic minority groups are particularly problematic because structural inequalities have generated an environment in which these groups are among the most vulnerable [17-23].

In this study, we analyzed a nationally representative survey of US adults conducted in late March 2020 to determine how race and ethnicity, and their intersections with gender and age, are associated with posting content related to COVID-19 on social media. Demographic intersections of age and gender with race and ethnicity are rarely inspected in studies characterizing social media users to understand potential biases in data sets [13,15]. However, it is particularly critical to examine these intersections during the COVID-19 pandemic because age and gender are additional fault lines shaping both social media use and the risk of experiencing direct and indirect effects of COVID-19. This analysis of digital divides can potentially offer guidance to researchers conducting public health surveillance studies who are concerned about biases in the social media data they are analyzing.

### Inequalities in Social Media Use and Effects of COVID-19

At present, little is known about how social media posting about COVID-19 varies across racial and ethnic groups. This is particularly problematic given the context of the COVID-19 pandemic. Within the United States, members of racial and ethnic minority groups experience heightened susceptibility to serious complications from COVID-19 because of underlying health and health care inequalities predating the pandemic [17-19]. Racial and ethnic minority groups are also overrepresented among essential workers, which elevates their risk of exposure to COVID-19 [21]. Further, they are more susceptible to indirect effects of the pandemic, such as job loss or pay cuts [20].

Digital divides raise questions about whether members of racial and ethnic minority groups are posting COVID-19 content on social media. Racial and ethnic minority groups in the United States have only recently made gains in internet access, narrowing the advantage held by white people [14,24]. Among people with internet access, members of racial and ethnic minority groups use social media at approximately the same rate as white people or more, depending on the platform [13,15,25]. Further, it has been suggested that the COVID-19 pandemic will exacerbate existing digital divides, due in part to the economic impacts of the pandemic and reduced access to public, workplace, and school internet connections [26]. The findings of public health surveillance tools leveraging social media data to track the COVID-19 pandemic may thus be biased toward underrepresenting the experiences of racial and ethnic minority groups.

Alternatively, members of racial and ethnic minority groups may be most likely to post COVID-19 content on social media. Given their higher risk of contracting COVID-19 and experiencing its deleterious effects, members of these groups are also more likely to struggle with mental health during the pandemic [23,27]. In response, they may turn to social media as a coping mechanism or to seek social support [28,29]. Social media data would then offer an opportunity for public health surveillance systems to understand racial and ethnic inequities during the pandemic.

Age is another axis of inequality for both social media use and complications arising directly and indirectly from COVID-19. Older adults (65 years and older) are less likely to access the internet and use it to share content on social media [30,31]. However, this demographic group was identified early in the pandemic as being at high risk for complications from COVID-19 [32] as well as for experiencing social isolation from mitigation efforts such as stay-at-home restrictions [33,34]. The intersection between race/ethnicity and age is also important because the older adult population is currently majority white but will become increasingly diverse in the next few years [35]. Public health surveillance tools relying on social media data must consider the impacts of these shifting demographics on digital divides.

Lastly, gender inequalities are also critical for identifying potential biases present in social media data. Women in the United States have outgained men in internet access [14,36]. Among internet users, women are generally more likely than men to use social media [13,15]; thus, it is probable that they are overrepresented in social media data. This digital divide in which women make greater use of social media may be problematic given that men appear to be at increased risk from COVID-19 complications [37]. Regarding the indirect effects of COVID-19, there are compelling reasons to expect distinct experiences for men and women. For example, men are more likely than women to rely on the workplace for confidants [38,39]. As a result, men who are working remotely during the pandemic, lose their job, or experience a reduction in work hours may have mental wellness impacts caused by diminished access to their usual sources of social support. By contrast, women are more likely to work as frontline nursing staff and to suffer indirect effects due to changing work patterns, increased exposure to intimate partner violence, and increased childcare obligations [40,41]. Race and ethnicity likely further modify these gender differences in experiences during the pandemic [42]; thus, an intersectional approach is required when analyzing which people are likely to post content on social media.

To date, no studies have considered how social media posting about COVID-19 differs by race and ethnicity, how these differences intersect with gender and age, or the implications of these patterns for public health surveillance efforts. Accordingly, the current study reports findings from a secondary analysis of a nationally representative survey fielded late March 2020.

## Methods

### Data Source

The data we analyzed were obtained from the Pew Research Center, a nonpartisan think tank that conducts regular surveys to track demographic trends. We analyzed the Wave 64 survey of their American Trends Panel, which is a probability-based online panel of US adults (people aged 18 years or older) [43]. Of the 15,433 invited panelists, 11,537 responded (74.8% response rate) in either English or Spanish from March 19 to 24, 2020. Among these panelists, 164/11,537 (1.4%) did not have an internet-enabled device but received an internet-enabled tablet to keep at no cost. The results shown are from analyzing 10,510 respondents, with complete responses on all measures used. Missing values for any measure were <1%, except for income, for which 3% of values were missing. An analysis using a multiple imputed data set (10 iterations) to fill in missing income values afforded results that are comparable to those shown here.

### Posting COVID-19–Related Content

The dependent measures were responses to an item asking whether they “used social media to share or post information about the coronavirus” (1=yes, 0=no).

### Race/Ethnicity, Gender, and Age

We measured these three demographics using the original groupings provided in the data set. Race and ethnicity were based on the respondent’s identification with one of four mutually exclusive categories (white, black, Latino, other). Gender was measured as a sex binary (female, male). Age was measured using four categories (18 to 29 years, 30 to 49 years, 50 to 64 years, 65 years or older).

### Covariates

Other measures associated with social media use and differential exposure to the direct and indirect effects of COVID-19 served as covariates in the multivariable analysis. The set of covariates directly measures experiences with economic hardship and struggles with mental health during the pandemic along with sociodemographic factors shaping differential exposure to these and other stressors, such as additional household labor and misinformation [13,15,29,30,44,45]. Covariates were respondents’ marital status (never married; currently married/cohabitating; divorced, widowed, or separated), annual family income (less than US $30,000, US $30,000-$74,999, greater than US $75,000), educational attainment (high school or less, some college, college graduate), political leaning (very liberal, liberal, moderate, conservative, very conservative), and mental health, which is an average of five items modified from the Center for Epidemiologic Studies Depression Scale [46] and General Anxiety Disorder Scale [47] asking how frequently (less than 1 day, 1 to 2 days, 3 to 4 days, 5 to 7 days) they experienced the following during the seven days preceding the survey (α=.73): nervous, anxious, or on edge; depressed; lonely; hopeful about the future; trouble sleeping. Higher values indicate poorer mental health. Household characteristics were census division, metropolitan area (yes, no), presence of a child less than 12 years of age (yes, no), presence of a household member who was laid off (yes, no), and presence of a household member who received a pay cut (yes, no). All groupings are original to the data set.

### Statistical Analysis

After describing the analytical sample, we present results from three multivariable logistic regressions estimating the odds that respondents would report that they used social media to post COVID-19 content. The first estimated the association of respondents’ race and ethnicity on the odds of posting content, adjusted for gender, age, and the covariates. The second examines how the intersection of race/ethnicity with gender shapes the odds of posting by estimating interactions between the racial and ethnic categories with gender. The estimated odds were adjusted for age and the covariates. The estimates were then used to compare the presence and severity of the gender divide by racial and ethnic group. The third regression followed a parallel procedure, enabling an assessment of the presence and severity of the age divide by racial and ethnic group. Specifically, we examined the intersection of race/ethnicity with age by estimating interactions between the racial and ethnic categories and the age groups. In these last two regressions, we corrected for making multiple comparisons.

All results shown (except for frequencies when describing the analytic sample) are based on weighting the data using the set of svy commands available in the statistical program Stata 16. The survey weights used in this process were provided by the Pew Research Center to reflect known US population counts on the following characteristics: age, race and ethnicity, gender, education, census region, metropolitan status, internet access, political party identification, volunteerism, registered voter, and years living in the United States.

## Results

Descriptive data for the analytical sample appear in Table 1. Means and percentages are survey-weighted, but frequencies reflect the unweighted sample. Of the respondents in the weighted sample, 4383/10,510 (39.4%) reported posting COVID-19 content on social media. The majority of the 10,510 respondents (7012, 65%) were white, and most (6700, 63.7%) were between 30 and 64 years of age. A slight majority of respondents were women (5724/10,510, 51%).

Table 2 shows the associations between respondents’ race and ethnicity and the odds of posting COVID-19 content on social media based on a multivariable logistic regression that adjusts for all covariates. Compared to the odds of posting for white respondents, black respondents had 29% higher odds (*P*=.03), Latino respondents had 66% higher odds (*P*<.001), and respondents identifying with other races or ethnicities had 33% higher odds (*P*=.03).

Table 2 also shows the independent associations of respondents’ gender and age with their odds of posting. Estimates depict a gender divide favoring women and an age divide favoring respondents aged 18 to 29 years. Women had 58% higher odds than men to report that they posted COVID-19 content (*P*<.001). Compared to respondents aged 18 to 29 years, respondents who are 65 and older were significantly less likely to report posting (*P*=.01).

The next two steps in the analysis examined the intersections between race/ethnicity and gender and age by estimating statistical interactions. Full estimation results are available in Multimedia Appendix 1. Here, we summarize key findings using figures plotting predicted probabilities and discrete changes in predicted probabilities, showing 95% confidence intervals around estimates.

**Figure 1:**
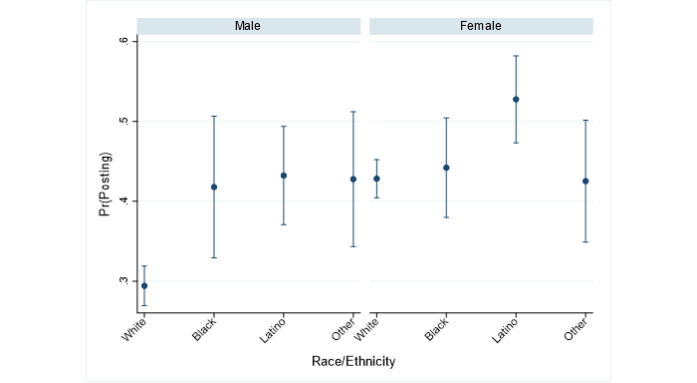
Predicted probabilities of posting (95% CI) for each gender by race and ethnicity. Pr(Posting): probability of posting.

**Figure 2:**
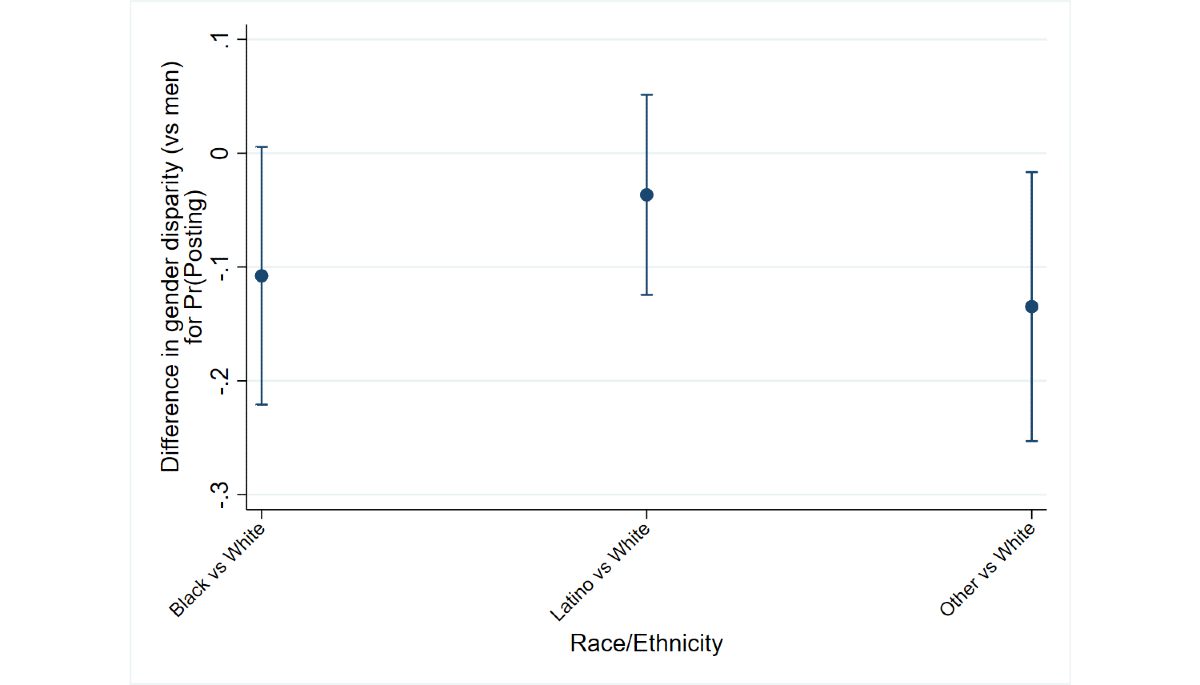
Differences in the gender divide in posting (95% CI) among respondents who identified as black, Latino, or other races/ethnicities relative to that of respondents who identified as white. Pr(Posting): probability of posting.

The first two figures summarize how the intersections of race and gender are associated with reports of posting COVID-19 content on social media. Figure 1 plots the predicted probabilities of posting COVID-19 content on social media for each cross-section. Among men, who were less likely to post (Table 2), respondents who identified as black (*P*=.009), Latino (*P*<.001), or other races/ethnicities (*P=*.003) were more likely to post than respondents who identified as white. Among women, only Latino respondents were significantly more likely to post than white respondents (*P*=.001). Figure 2 summarizes how the severity of the gender divide in posting COVID-19 content among respondents who identified as black, Latino, or other races/ethnicities compares to that of respondents who identified as white. Values crossing zero indicate comparable severity, while negative values indicate an attenuation of the divide. The figure shows a tendency for the gender divide to be slightly narrower among respondents identifying as either black (*P*=.06) or other races (*P*=.03); these findings did not hold when adjusting for multiple comparisons.

**Table 1:** Characteristics of sample respondents (N=10,510).

Characteristic		Value
Posted COVID-19^a^ content on social media, n (%)		4383 (39.4)
**Race/ethnicity, n (%)**		
	White	7012 (65.4)
	Black	771 (10.3)
	Latino	2145 (15.6)
	Other	582 (8.7)
Female gender, n (%)		5724 (51.3)
**Age (years), n (%)**		
	18-29	1219 (20.9)
	30-49	3569 (35.4)
	50-64	3131 (24.6)
	≥65	2591 (19.1)
**Annual family income (US $), n (%)**		
	<30,000	1897 (27.7)
	30,000-74,999	3618 (36.8)
	≥75,000	4995 (35.4)
**Education, n (%)**		
	High school or less	1397 (33.8)
	Some college	3141 (32.5)
	College graduate	5972 (33.7)
Household member was laid off, n (%)		1833 (19.5)
Household member received pay cut, n (%)		2776 (27.4)
Mental health^b^, mean (SD)		2.07 (0.72)
US citizen, n (%)		9999 (93.4)
**Marital status, n (%)**		
	Never married	1823 (17.4)
	Currently married or cohabitating	6787 (58.2)
	Divorced, widowed, or separated	1900 (17.3)
Young child (<12 years) in household		2370 (24.9)
**Political leaning, n (%)**		
	Very liberal	1030 (8.1)
	Liberal	2275 (17.7)
	Moderate	4074 (41.4)
	Conservative	2347 (24.4)
	Very conservative	784 (8.5)
	In metropolitan area	9435 (87.2)
**Census division, n (%)**		
	Pacific	1491 (14.5)
	Middle Atlantic	1158 (12.6)
	East North Central	1467 (14.7)
	West North Central	689 (6.5)
	South Atlantic	2966 (21.4)
	East South Central	458 (5.0)
	West South Central	996 (10.9)
	Mountain	817 (9.2)
	New England	468 (5.1)

^a^COVID-19: coronavirus disease.

^b^Average of five items modified from the Center for Epidemiologic Studies Depression Scale and General Anxiety Disorder Scale. Higher values indicate poorer mental health.

**Table 2:** Associations between respondent characteristics and reported posting on social media about the COVID-19 pandemic.

Characteristic		Odds ratio (95% CI)		*P* value	
**Race/ethnicity**					
	White		Reference		N/A^a^
	Black		1.29^b^ (1.02-1.64)		.04
	Latino		1.66^c^ (1.36-2.04)		<.001
	Other		1.33^b^ (1.02-1.72)		.03
**Gender**					
	Male		Reference		N/A
	Female		1.58^c^ (1.39-1.80)		<.001
**Age (years)**					
	18-29		Reference		N/A
	30-49		0.99 (0.80-1.23)		.94
	50-64		0.95 (0.75-1.20)		.67
	≥65		0.73^b^ (0.57-0.94)		.01
**Annual family income (US $)**					
	<30,000		Reference		N/A
	30,000-74,999		0.98 (0.82-1.18)		.86
	≥75,000		0.82^b^ (0.67-0.99)		.04
**Education**					
	High school or less		Reference		N/A
	Some college		1.12 (0.94-1.33)		.20
	College graduate		1.10 (0.92-1.30)		.29
Household member was laid off		1.17 (0.97-1.41)		.10	
Household member received pay cut		1.05 (0.89-1.23)		.57	
Mental health^d^		1.27^c^ (1.15-1.40)		<.001	
US citizen		0.69^b^ (0.49-0.97)		.03	
**Marital status**					
	Never married		Reference		N/A
	Currently married or cohabitating		1.16 (0.96-1.40)		.13
	Divorced, widowed, or separated		1.18 (0.94-1.47)		.15
Young child (<12 years) in household		1.18 (1.00-1.41)		.06	
**Political leaning**					
	Very liberal		Reference		N/A
	Liberal		0.80 (0.62-1.01)		.07
	Moderate		0.66^c^ (0.52-0.83)		<.001
	Conservative		0.77^b^ (0.6-0.99)		.045
	Very conservative		0.70^b^ (0.51-0.96)		.03
In metropolitan area		1.01 (0.82-1.25)		.90	
**Census division**					
	Pacific		Reference		N/A
	Middle Atlantic		1.29^b^ (1.01-1.64)		.04
	East North Central		1.21 (0.95-1.53)		.12
	West North Central		1.03 (0.77-1.38)		.85
	South Atlantic		1.21 (0.98-1.50)		.07
	East South Central		1.45^b^ (1.04-2.03)		.03
	West South Central		1.11 (0.85-1.44)		.44
	Mountain		0.98 (0.73-1.30)		.87
	New England		1.27 (0.91-1.77)		.16

^a^N/A: not applicable.

^b^*P*<.05.

^c^*P*<.001.

^d^Average of five items modified from the Center for Epidemiologic Studies Depression Scale and General Anxiety Disorder Scale. Higher values indicate poorer mental health.

**Figure 3:**
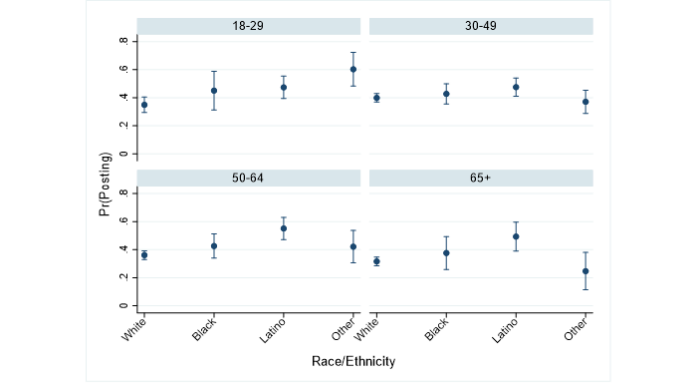
Predicted probabilities of posting (95% CI) for each age group by race and ethnicity. Pr(Posting): probability of posting.

The remaining two figures offer a parallel summary of the intersections between the race of the respondents and their ethnicity and age. Figure 3 shows the predicted probabilities of posting for all four age groups by race and ethnicity of the respondents. Latino respondents were significantly more likely than white respondents across all age groups to report posting COVID-19 content on social media (18 to 29 years: *P*=.01, 30 to 49 years: *P*=.04, 50 to 64 years: *P*<.001, ≥65 years: *P=*.001). While respondents aged 65 years or older were less likely to post COVID-19 content than respondents aged 18 to 29 years (Table 2), those who did post content were likely to identify as Latino. The only other significant finding from this analysis was that among respondents aged 18 to 29 years, respondents identifying as a race/ethnicity other than black, white, or Latino had a higher probability of posting than white respondents (*P*<.001).

**Figure 4:**
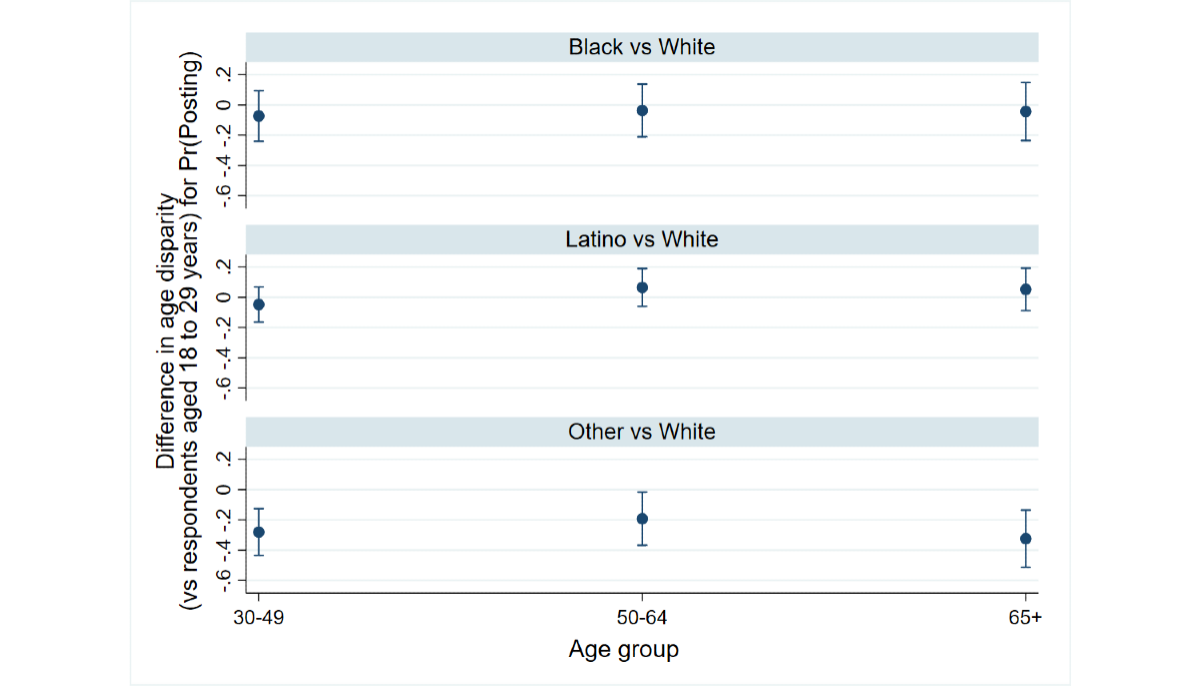
Differences in the age divide in posting (95% CI) among respondents who identified as black, Latino, or other races/ethnicities relative to respondents who identified as white. Pr(Posting): probability of posting.

Figure 4 continues the summary of the intersections between race and age by displaying the additional contributions of race and ethnicity to the age divide among respondents who post COVID-19 content on social media. The figure shows how the differences in the predicted probability between respondents aged 18 to 29 years and each of the older age groups change for respondents who identified as black, Latino, or other races/ethnicities relative to respondents who identified as white. A value crossing zero indicates comparable predicted differences in probability, with positive values indicating a wider age divide among the group compared to white respondents. Age divides were significantly intensified among respondents identifying as other races (31 to 49 years: *P*<.001, 50 to 64 years: *P*=.04, ≥65 years: *P=*.001), perhaps because of the heightened tendency of respondents aged 18 to 29 years from this racial/ethnic group to post (Figure 1). When adjusting for multiple comparisons, these conclusions held except among respondents aged 50 to 64 years. The severity of the age divide was comparable in all other cases.

## Discussion

### Principal Findings

US public and public health surveillance analysts are both turning to social media during the COVID-19 pandemic. Specifically, members of the public are posting content related to the pandemic; in turn, analysts are using this content to track the spread of the virus and its effects across the population. However, racial and ethnic digital divides raise questions about the representativeness of the social media data that analysts are using to make inferences and projections. We found that among a nationally representative sample of US adults, members of racial and ethnic minority groups were most likely to report posting COVID-19 content on social media. Further, among men and older adults, who are traditionally less likely to use social media, members of racial and ethnic minority groups were also most likely to report posting content. Studies like this one that examine digital divides are critical to understanding the characteristics of people posting content to better infer the presence of biases in social media data. Other steps are likely necessary to fully address issues of representativeness in data, such as ensuring that data collection processes and search criteria are designed to capture diversity in the data.

Respondents who identified as black, Latino, or other races and ethnicities were more likely to report posting COVID-19 content on social media than respondents who identified as white. This finding may simply be an artifact of a reversal of digital divides, whereby white people are now least likely to use social media. A reversal is possible given trends observed during February 2019 in the United States [25]. While the estimated racial and ethnic differences from February 2019 cannot adequately account for the odds ratios in the current study, it is difficult to rule out this possibility without a demographic profile of social media users during the days preceding the pandemic. Alternatively, members of racial and ethnic minority groups may be most likely to post because the specific pandemic context (in which they are at higher risk of direct and indirect effects of COVID-19) inspires a stronger motivation to post. Specifically, our model controlled for financial ramifications from COVID-19 and mental health; however, there may be other COVID-19–related factors driving posting. Uses and gratifications theory suggests that social media use is often driven by a desire for self-expression, self-documentation, and social connection [48]. Social media also offers a space for seeking social support and coping with stress [28,29]. Although there may be culturally driven distinctions in posting behaviors related to uses and gratifications, there is little prior research considering how these distinctions differ across race and ethnicity [49]. More work is needed to understand differences in posting behaviors, particularly to better understand COVID-19.

Regardless of the reason that members of racial and ethnic minority groups are most likely to post about COVID-19 on social media, our findings suggest that public health surveillance tools relying on social media data may be well positioned to capture their pandemic experiences and how they are related to structural inequalities within the United States. It will be critical for researchers to continue to both reflect on the overall representativeness of social media data and consider the nuances of social media use and data collection strategies when seeking data on specific populations. For example, a higher proportion of the Latino population than the white population uses Instagram, while these rates are more consistent for Facebook [25].

The intersections with gender and age were also enlightening for understanding digital divides during the pandemic. Women are generally more likely than men to use social media [13,15], and we saw this divide reflected across all racial and ethnic groups with respect to posting about COVID-19. Among men, those in racial and ethnic minority groups were most likely to report posting. These racial and ethnic differences are important to bear in mind depending on which gender disparities in COVID-19 experiences researchers seek to understand. With respect to age, studies consistently show that older adults are less likely to use social media [30], which was reflected in the findings of this study. This is important because of the elevated risks older adults face during the pandemic. Among respondents 65 years or older, Latino respondents are significantly more likely to post, suggesting an opportunity to leverage their social media data to understand their experiences. Such intersections with race and ethnicity are rarely investigated in studies on digital divides.

### Limitations

This study is not without limitations. The dependent measure (posting content on social media related to COVID-19) is self-reported; thus, it is susceptible to recall bias. However, the survey was administered shortly after the outbreak occurred in the United States, which mitigates some of the potential bias. The measure also does not allow us to determine the content of posts or which social media platforms were used. Previous work showing racial and ethnic demographic variation by platform can offer some guidance [15]. Moreover, we cannot distinguish between users who post original content versus users who repost others’ content (eg, a retweet on Twitter). Other datasets combining a user’s social media data with their demographic profile are better suited for identifying users who are likely to repost [44]. Limitations such as these are common within studies relying on a secondary analysis of nationally representative data.

### Conclusions

Despite limitations, the results of this examination of digital divides offer key insights into who is contributing to the social media content related to COVID-19 that analysts are studying. Because COVID-19 is exacerbating previously existing inequalities in the United States, members of racial and ethnic minority groups experience elevated risk of experiencing the deleterious health and economic effects of the virus. These populations are also most likely to post COVID-19–related content. There is also evidence of a heightened tendency to post among members of racial and ethnic minority groups who are members of other vulnerable groups that are usually less likely to use social media (people aged 65 years and older, men). These patterns suggest that social media data offer opportunities for public health researchers seeking to understand the impacts and spread of COVID-19 among racial and ethnic minority groups in the United States. We further suggest that it is not sufficient for researchers to rely only on broad social media usage demographics when reflecting on the representativeness of their data, as COVID-19 posting does not perfectly mirror what would be anticipated based on usage statistics alone [25].

To ensure that public health surveillance efforts take digital divides into account, studies like this one are a critical first step in that they provide demographic context for user-generated data. Additional steps are needed to realize this opportunity fully and mitigate bias. For example, collecting data in several languages, including Spanish, may ensure that the data reflect the patterns observed here among Latino respondents. A publicly available dataset of tweets associated with the pandemic is currently being collected [7]. The data collection process must incorporate a breadth of social media platforms and ensure that search criteria are reflective of the linguistic diversity in discourse about COVID-19. Given that the selection of search criteria shapes social media research findings [50], it is also critical for future research to map language use and how it differs across demographics. These steps are critical to meeting the challenges presented by the COVID-19 pandemic and understanding its lasting effects on the US population.

## Appendix

Multimedia Appendix 1 Estimates for multivariable logistic regressions.

## Abbreviations

COVID-19: coronavirus disease
