# Supplementary material for: Racial and Ethnic Digital Divides in Posting COVID-19 Content on Social Media Among US Adults: Secondary Survey Analysis
Source: J Med Internet Res. 2020 Jul 3;22(7):e20472. doi: 10.2196/20472 (PMC7340161; doi:10.2196/20472)
Supplement: Multimedia Appendix 1 [file jmir_v22i7e20472_app1.docx]

| Table S1. Odds Ratios of Posting on Social Media about the COVID-19 Pandemic with Interactions between Race/Ethnicity and Gender | |
| --- | --- |
| Characteristic | Odds Ratio (95% CI) |
| Race/ethnicity (White) |  |
| Black | 1.75^b^ (1.18-2.60) |
| Latino | 1.86^c^ (1.38-2.49) |
| Other | 1.82^b^ (1.25-2.65) |
| Female (Male) | 1.83^c^ (1.57-2.12) |
| Black x Female | 0.61^a^ (0.38-0.97) |
| Latino x Female | 0.81 (0.56-1.18) |
| Other x Female | 0.54^a^ (0.33-0.89) |
| Age (18-29) |  |
| 30-49 | 0.99 (0.80-1.23) |
| 50-64 | 0.95 (0.76-1.20) |
| 65+ | 0.73^c^ (0.57-0.93) |
| Annual family income (<$30,000) |  |
| $30-74,999 | 0.98 (0.81-1.18) |
| $75,000+ | 0.81^a^ (0.67-0.99) |
| Education (High school or less) |  |
| Some college | 1.12 (0.94-1.33) |
| College graduate | 1.09 (0.92-1.29) |
| Household member laid off | 1.16 (0.97-1.40) |
| Household member with pay cut | 1.05 (0.89-1.23) |
| Mental health | 1.27^c^ (1.15-1.40) |
| U.S. Citizen | 0.71^a^ (0.50-0.99) |
| Marital status (Never married) |  |
| Current married or cohabitating | 1.16 (0.95-1.40) |
| Divorced, widowed, or separated | 1.17 (0.94-1.46) |
| Young child (< 12yrs) in household | 1.19 (1.00-1.41) |
| Political leaning (Very liberal) |  |
| Liberal | 0.80 (0.63-1.02) |
| Moderate | 0.66^c^ (0.52-0.83) |
| Conservative | 0.78 (0.61-1.00) |
| Very conservative | 0.70^b^ (0.51-0.96) |
| In metropolitan area | 1.02 (0.83-1.26) |
| Census division (Pacific) |  |
| Middle Atlantic | 1.27 (1.00-1.63) |
| East North Central | 1.21 (0.96-1.54) |
| West North Central | 1.02 (0.76-1.37) |
| South Atlantic | 1.20 (0.98-1.49) |
| East South Central | 1.46^a^ (1.05-2.04) |
| West South Central | 1.12 (0.86-1.46) |
| Mountain | 0.98 (0.74-1.31) |
| New England | 1.26 (0.90-1.76) |
| ^a^ *P* < .05 ^b^ *P* < .01 ^c^ *P* < .001 | |
| Table S2. Odds Ratios of Posting on Social Media about the COVID-19 Pandemic with Interactions between Race/Ethnicity and Age | |
| Characteristic | Odds Ratio (95% CI) |
| Race/ethnicity (White) |  |
| Black | 1.56 (0.83-2.91) |
| Latino | 1.71^a^ (1.14-2.59) |
| Other | 2.95^c^ (1.66-5.24) |
| Female (Male) | 1.59^c^ (1.39-1.80) |
| Age (18-29) |  |
| 30-49 | 1.25 (0.94-1.65) |
| 50-64 | 1.05 (0.79-1.41) |
| 65+ | 0.86 (0.64-1.16) |
| Black x 30-49 | 0.73 (0.36-1.46) |
| Black x 50-64 | 0.85 (0.41-1.78) |
| Black x 65+ | 0.85 (0.37-1.92) |
| Latino x 30-49 | 0.81 (0.49-1.33) |
| Latino x 50-64 | 1.31 (0.76-2.24) |
| Latino x 65+ | 1.27 (0.69-2.31) |
| Other x 30-49 | 0.30^b^ (0.15-0.59) |
| Other x 50-64 | 0.44^a^ (0.21-0.95) |
| Other x 65+ | 0.24^b^ (0.09-0.61) |
| Annual family income (<$30,000) |  |
| $30-74,999 | 0.96 (0.80-1.16) |
| $75,000+ | 0.79^a^ (0.65-0.96) |
| Education (High school or less) |  |
| Some college | 1.12 (0.94-1.33) |
| College graduate | 1.10 (0.93-1.31) |
| Household member laid off | 1.17 (0.97-1.40) |
| Household member with pay cut | 1.05 (0.89-1.24) |
| Mental health | 1.26^c^ (1.15-1.40) |
| U.S. Citizen | 0.67^a^ (0.48-0.94) |
| Marital status (Never married) |  |
| Current married or cohabitating | 1.16 (0.96-1.41) |
| Divorced, widowed, or separated | 1.17 (0.94-1.46) |
| Young child (< 12yrs) in household | 1.20^a^ (1.01-1.42) |
| Political leaning (Very liberal) |  |
| Liberal | 0.80 (0.62-1.02) |
| Moderate | 0.66^c^ (0.52-0.83) |
| Conservative | 0.77^a^ (0.60-1.00) |
| Very conservative | 0.69^a^ (0.50-0.96) |
| In metropolitan area | 1.02 (0.83-1.25) |
| Census division (Pacific) |  |
| Middle Atlantic | 1.28^a^ (1.00-1.64) |
| East North Central | 1.19 (0.94-1.51) |
| West North Central | 1.04 (0.78-1.39) |
| South Atlantic | 1.20 (0.97-1.49) |
| East South Central | 1.48^a^ (1.07-2.06) |
| West South Central | 1.12 (0.86-1.45) |
| Mountain | 0.99 (0.74-1.32) |
| New England | 1.28 (0.91-1.79) |
| ^a^ *P* < .05 ^b^ *P* < .01 ^c^ *P* < .001 | |
